# How Changes in Reimbursement Practices Influence the Financial Sustainability of Medicine Policy: Lessons Learned from Slovakia

**DOI:** 10.3389/fphar.2019.00664

**Published:** 2019-06-13

**Authors:** Tomas Tesar, Branislav Obsitnik, Zoltán Kaló, Finn Børlum Kristensen

**Affiliations:** ^1^Department of Organisation and Management in Pharmacy, Faculty of Pharmacy, Comenius University in Bratislava, Slovakia; ^2^St. Elisabeth’s Institute of Oncology, Bratislava, Slovakia; ^3^Syreon Research Institute, Budapest, Hungary; ^4^Department of Health Policy and Health Economics, Eötvös Loránd University (ELTE), Budapest, Hungary; ^5^Research Unit of User Perspectives, Department of Public Health, Faculty of Health Sciences, University of Southern Denmark, Odense, Denmark

**Keywords:** technology assessment, balanced assessment, decision-making, insurance, reimbursement, health policy, Slovakia

## Abstract

**Objectives:** The aim of this study was to review the impact of new reimbursement requirements for medicines in the Slovak Republic based on legislation that came into force in January 2018.

**Methods:** The new legislation was reviewed. The reimbursement dossiers for medicines and health technology assessments and appraisals, justifications for reimbursement decisions, final reimbursement decisions, and all aspects of the appeal mechanisms have been transparently published on the website of the Slovak Ministry of Health and were used for this analysis.

**Results:** Based on the new legislation, there was no need to submit information about relative effectiveness and cost-effectiveness of medicines with less than 1:50,000 eligible patients prior to reimbursement decisions, and the cost-effectiveness threshold has been increased for all other medicines. The estimated impact of the 2-year budget for the 59 medicines submitted for reimbursement without relative effectiveness and cost-effectiveness analysis was €181,273,698, based on the published submission dossiers. The estimated impact of the 2-year budget for the 45 medicines with evidence of relative effectiveness and cost-effectiveness was €178,566,634. In contrast to the easier market access criteria for new original medicines, the new legislation enforces stricter price erosion criteria for generic and biosimilar medicines. Consequently, the number of generic and biosimilar entries was reduced from 242 in 2017 to 224 in 2018.

**Conclusions:** Although some of the new reimbursement applications were not approved by the Ministry of Health, many new medicines were added to the Slovak pharmaceutical reimbursement list based on “balanced assessment” requirements; hence, the system became financially unsustainable. It was necessary to change the legislation from January 2019.

## Introduction

This article reports on selected legislative initiatives and their implementation, focusing on the consequences for financial sustainability of the Slovak health care system. The universal health coverage system in Slovakia is based on mandatory health insurance with a basic benefit package provided by competing health insurance companies, and selective contracting of health care providers and flexible pricing of health services (Smatana et al., 2016).

The strategic pricing of innovative medicinal products is not based on the needs of small markets with low purchasing power (such as Slovakia), as pharmaceutical companies adjust the price level of their new drugs to the requirements of wealthier countries with a greater willingness to pay for one unit of health gain (Kaló et al., 2013). In lower-income countries, relative effectiveness and cost-effectiveness analyses can help decision-makers to judge the local value of new technologies. Slovakia had not utilized cost-effectiveness analyses to support reimbursement decisions before 2011; thus, several pharmaceuticals that were potentially not cost-effective had been reimbursed (Kaló et al., 2008).

In 2011, Act 363/2011 (Ministry of Health, 2011) mandated new rules for external price referencing and pharmacoeconomic evaluation in the pricing and reimbursement of medicinal products. The “maximum retail price” (ex-factory price) of medicines could not exceed the average of the three lowest prices of the same medicine across the European Union (EU) (Tesar et al., 2017). Calculation of the relative effectiveness of new medicines (preferably in QALYs) to a policy relevant comparator, the impact of new medications on the public health insurance budget, and evaluation of cost-effectiveness were also mandated. The Reimbursement Committee determines the therapeutic and social value of the medicine based on criteria summarized in **Table 1**. Calculation of the threshold value coefficient was determined by Act 363/2011, and the lower (λ1) and upper (λ2) cost-effectiveness thresholds were defined as 24 and 35 times the average monthly salary, respectively. This meant that the threshold could be increased automatically in positive economic periods. **Table 2** indicates the development of cost-effectiveness thresholds in the Slovak Republic.

**Table 1:** Criteria determining the therapeutic and social value of medicines (Szalay et al., 2011).

Criteria determining the therapeutic value of medicines	Criteria determining the social value of medicines
Relative effectiveness (i.e., QALY gain)	Severity of the disease
Safety	Impact on society if not treated (e.g., spread of infection)
Cost-effectiveness	Social value (e.g., orphan drugs)
Whether it is a first or second option or adjunctive treatment	Risk of abuse
Whether it is a causal, prophylactic or symptomatic treatment	Impact on total costs

**Table 2:** Cost-effectiveness thresholds in the Slovak Republic.

Before 2011	Without cost-effectiveness thresholds	Kaló et al., 2008
Between 2011 and 2017	Lower (λ1) and upper (λ2) cost-effectiveness thresholds were defined as 24 and 35 times the average monthly salary	Act. 363/2011 came into force from December 01, 2011
From 2018	Lower (λ1) and upper (λ2) cost-effectiveness thresholds were defined as 35 and 41 times the average monthly salary	Updated Act. 363/2011 came into force from January 01, 2018

Van Wilder et al. (2015) concluded that in Slovakia, thresholds for additional costs per quality-adjusted life year (QALY) were used as a tool to assess the cost-effectiveness of medicines, and not as a rule by which medicines were excluded from consideration for reimbursement. Owing to the cost-effectiveness criterion, several highly priced medicines could not be directly added to the positive drug list in Slovakia; however, Act. 363/2011 allowed payers to fully or partially reimburse medicines that were not cost-effective on an exceptional basis (Bucek Psenkova et al., 2017). Reimbursement exemptions by payers of medicines that were not cost-effective could be issued based on specific patient access schemes for certain innovative medicines. Such schemes were negotiated directly between pharmaceutical manufacturers and health insurance companies. Usually, price discounts or price caps were offered in exchange for public reimbursement.

According to Barnieh et al. (2014), the Slovak Republic met the highest standard of the three criteria deemed important in the process of pharmaceutical reimbursement, including consideration of clinical and cost evidence, full transparency, and the presence of a formal appeal mechanism. Still, concerns were raised relating to the impact of explicit cost-effectiveness thresholds on patient access to very expensive products (Psenkova et al., 2014).

**Figure 1:**
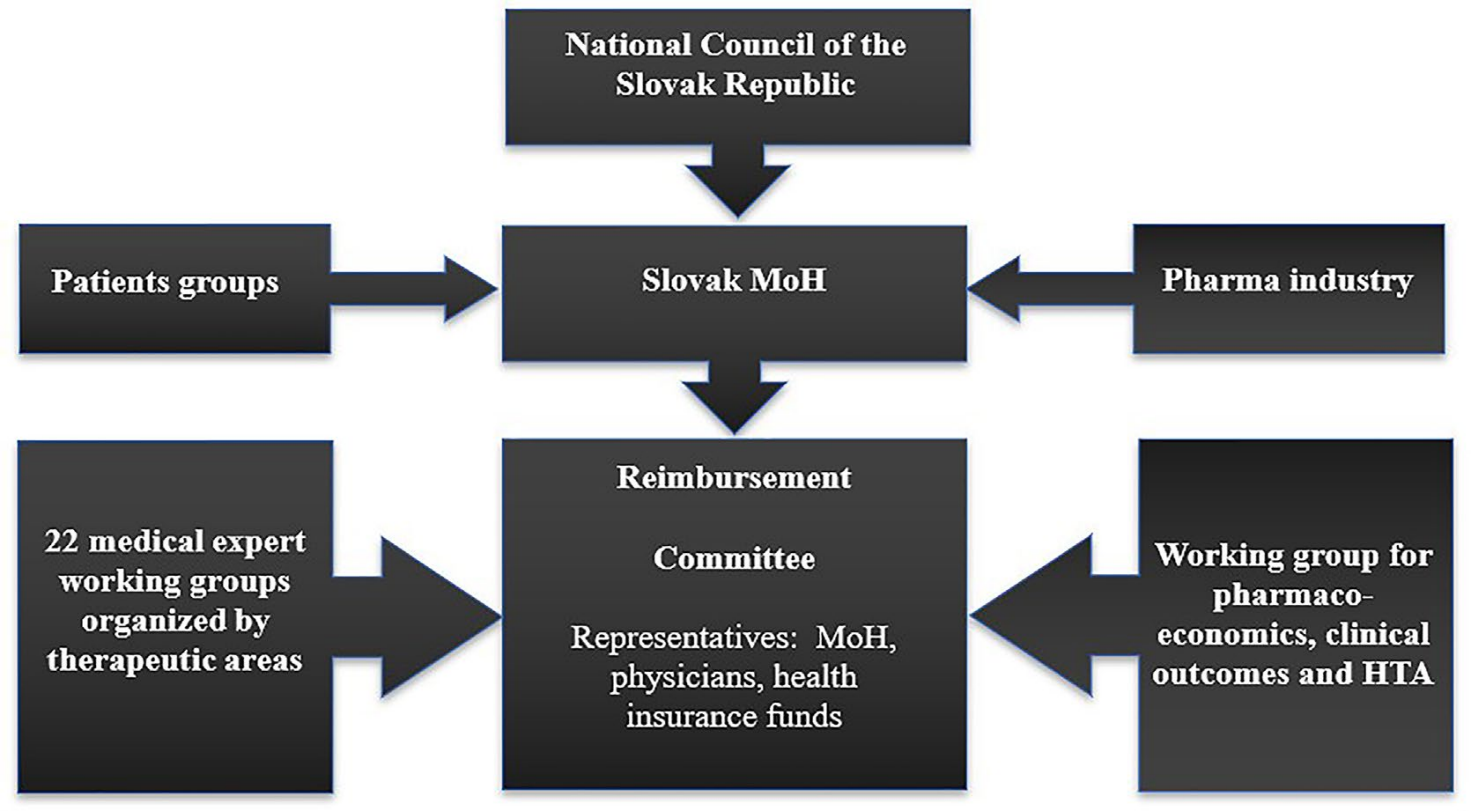
Reimbursement process for pharmaceuticals in Slovakia.

The Slovak Ministry of Health established the Reimbursement Committee to act as its advisory body for reimbursement processes. The Committee comprises three representatives from the Ministry of Health, three representatives from the Slovak Medical Chamber, and five representatives from health insurance companies. The Committee is supported by advisory working groups, which comprise medical boards and the Working group for Pharmacoeconomics, Clinical Outcomes, and Health Technology Assessment (HTA), as shown in **Figure 1**.

The Committee prepares recommendations for reimbursement levels, patient co-payments, and conditions for reimbursement. Based on the recommendations from the Reimbursement Committee, the Minister of Health issues final decisions. In addition, in January 2017, a Health Technology Assessment Department was established at the Slovak Ministry of Health with limited human resource capacities.

In recent years, several events have been organized to promote the rationale for an increased cost-effectiveness threshold and a simpler market access pathway for medicines that lack a cost-effectiveness analysis, according to a “balanced assessment” (Dankó, 2014). The new legislative framework, which came into force on January 1, 2018, significantly changed the pharmaceutical pricing and reimbursement procedures related to cost-effectiveness thresholds in the Slovak Republic.

## Materials and Methods

We reviewed the legislation, including updated Act 363/2011 (which came into force on January 1, 2018) regarding the scope and conditions of payments for medicines, medical devices, and dietetic foods from public health insurance, and Decree 93/2018 regarding the criteria for determining the significance of the effect of a medication on the public health insurance funds budget; the evaluation criteria for the calculation of the threshold value coefficient; and details of the calculation of the threshold value coefficient. In addition, we reviewed and analyzed the reimbursement dossiers for medicines, HTAs and appraisals, the justifications for reimbursement decisions, the final reimbursement decisions, and all aspects of the appeal mechanisms in 2018, which have been transparently published on the website of the Slovak Ministry of Health1.

## Results

According to the updated Act 363/2011 (Ministry of Health, 2011), in 2018, Slovakian lower and upper cost-effectiveness thresholds to support reimbursement decisions were increased to 35 times the average monthly salary (λ_1_ = €31,920/QALY) and 41 times the average monthly salary (λ_2_ = €37,392/QALY), respectively. **Table 3** indicates that in 2018, the upper cost-effectiveness threshold in Slovakia was higher than those in Poland and Hungary. With an increasing average monthly salary, the basic (λ_1_) and maximal (λ_2_) thresholds were increased to €33,390/QALY and €39,114/QALY, respectively, in 2019.

**Table 3:** Cost-effectiveness thresholds in Poland, Hungary, Slovakia, and England in 2018.

	Poland	Hungary	Slovakia	England
Lower threshold (€/QALY)*	–	–	31,920	22,606
Upper threshold (€/QALY)*	32,841	36,890	37,392	33,909 (56,516**; 113,030***)

*2018 annual exchange rate: 318.89 HUF/€; 4.2615 PLZ/€; 0.88471 GBP/€ (see https://ec.europa.eu/eurostat/web/exchange-and-interest-rates/data/main-tables, accessed on 24 Feb 2019).

**Special threshold for end-of-life therapies.

***Special threshold for orphan medicines.

QALY, quality-adjusted life year.

In general, medicines can be reimbursed by public health insurance (fully or partially) if the incremental cost per incremental QALY is not higher than λ1. In specific cases stated in Decree 93/2018 (Ministry of Health, 2018), the thresholds per incremental QALY can be increased up to λ2 or decreased to below λ1. The basic threshold (λ1) can be changed based on multiple criteria, including 1) recommendations from HTA bodies or reimbursement status in three important European countries (Germany, France, and the United Kingdom), 2) the availability of alternative medicines, 3) the budget impact, 4) the magnitude of health gain in QALYs, and 5) orphan designation of the new medicine.

In accordance with the Act, if all four HTA agencies in France, Germany, Scotland, and England give positive recommendations or when the medicine has already been reimbursed in France, Germany, Scotland, and England, the basic threshold should be applied. If none of the four HTA agencies in France, Germany, Scotland, or England have made any positive recommendations or the medicinal product has not been reimbursed in any of these countries, then the threshold has to be reduced to 34 times the average monthly salary.

In those cases when five or more medicines are available for the same indication on the national reimbursement list, a reduced threshold (34 times the average monthly salary) should be applied. In those cases when no other medicine is available with the same indication on the national reimbursement list, an increased threshold (36 times the average monthly salary) is applicable. In those cases when the 1-year budget impact of the new medicine is higher than €4,001,000, a reduced threshold (32 times the average monthly salary) is applicable. In those cases when the 1-year budget impact of the medicine is not greater than €100,000, an increased (36 times the average monthly salary) threshold is applicable.

In those cases when the medicine is not expected to deliver 0.1 QALYs per patient according to the calculation by the manufacturer in the submitted reimbursement dossiers, a reduced threshold (33 times the average monthly salary) is applicable. In those cases when the medicine is expected to deliver at least 2.1 QALYs, the threshold becomes 37 times the average monthly salary.

If the medicine has orphan designation, an increased threshold (37 times the average monthly salary) is applicable. The combination of all factors for threshold increases can result in the application of a maximal threshold (λ_2_), which is 41 times the average monthly salary (total €39 114/QALY).

From 2018, pharmaceutical manufacturers have not been mandated to attach a pharmacoeconomic analysis to the reimbursement dossier, if the medicine was aimed to treat a disease for which the number of eligible patients based on the indication approved in the marketing authorization in the Slovak Republic was lower than 1:50,000. Overall, manufacturers of 59 medicines (some of them with several different doses or package sizes) submitted application to the national reimbursement system without information on relative effectiveness (i.e., QALY gain) and cost-effectiveness. According to the estimates by pharmaceutical companies, the 2-year budget impact of these medicines was €181,273,698 (see **Supplement 1**). The estimated 2-year budget impact of other 45 pharmaceutical submissions (again, with different doses and package) with relative effectiveness and cost effectiveness evidence (compared to the increased thresholds) was €178,566,634.

The true budgetary impact of newly reimbursed medicines remains unclear for two reasons. Firstly, not all reimbursement applications were approved by the Ministry of Health, often because the Ministry of Health did not agree that the medicines were aimed to treat a disease with less than 1:50,000 eligible patients. Secondly, the Ministry of Health maximized the available budget for some newly reimbursed medicines at a lower level compared to the budget impact calculations by the manufacturer in the reimbursement dossier. On the other hand, confidential price reduction was not applied in pharmaceutical reimbursement decisions by the Ministry of Health discount agreements in 2018.

The utilization of generic and biosimilar medicines can support the financial sustainability of the pharmaceutical budget. In order to facilitate price erosion after the patent expiry of original medicines, the 2018 legislation introduced mandatory discounts for off-patent medicines. According to the new law, the first generic medicine entering the Slovak market must have a 45% initial price reduction compared to the original medicine, the second generic must have an additional 10% price reduction compared to the first, and the third generic must have an additional 5% price reduction compared to second. This three-step system for generic medicines is also used in other European countries (Vogler, 2012). The rule of a mandatory 10% or 5% price reduction was applied to the second and third generics only after an external price referencing procedure. This means that these products could not be priced higher than the average of the three lowest prices of the same medicine in other EU countries. If this criterion is fulfilled, they have to be 10% or 5% cheaper than the previously launched generic medicine.

The legislation resulted in similar changes to biosimilar medicines. The first biosimilar entering the Slovak market in 2018 must have a 30% initial price reduction compared to the price of the original biologic medicine, the second biosimilar must be launched with an additional 5% price reduction compared to the first biosimilar, and the third biosimilar must have an additional 5% price reduction compared to the second biosimilar. The rule of a mandatory 5% price reduction was applied to the second and third biosimilar only after an external price referencing procedure, similar to the external price referencing for generic medicines referred to above.

The domino effect of external price referencing has implications in two directions. In addition to the influence of other countries on generic and biosimilar drug prices in Slovakia, the Slovakian prices of generic and biosimilar medicines may also induce global price erosion of generics and biosimilars in other markets. Consequently, market access criteria of the second and third off-patent medicines have become fairly strict, which has prevented some manufacturers from launching products in the Slovakian market.

The three-step system for reducing prices was also applied to new-package versions of generics and biosimilars. For example, when the package size was changed from 10 to 30 tablets, it was also considered a new generic or biosimilar product, which also complicated the launch of off-patent medicines with new package sizes. In contrast, this rule has not been applied to the original products. Thus, within the same reference group, two competing products can set very different conditions for the introduction of simple differences in package size.

Overall, the new legislation is probably among the contributing factors, which have decreased the frequency with which generics and biosimilars have been launched in Slovakia. Compared to the 242 submissions in 2017, only 224 dossiers of generic and biosimilar medicines were submitted for reimbursement in 2018.

## Discussion

Slovak pharmaceutical spending per capita is in line with the EU-28 average and exceeds the corresponding values for several countries with better health outcomes (OECD Health at a Glance, 2018). For example, the Czech Republic, Denmark, and Slovenia have lower per capita pharmaceutical spending and lower amenable mortality, suggesting that money spent on medicines in Slovakia could be spent more efficiently on other health care services (Dancikova et al., 2018). This also indicates that increased market access for new medicines may not necessarily be a top priority in reforming the Slovak health care system.

Even if increased market access for new medicines is prioritized by healthcare policymakers, it is questionable whether an increased cost-effectiveness threshold and a simpler market access pathway based on a “balanced assessment” are appropriate tools to achieve this objective. It is difficult to identify any sources for the scientific validation of the “balanced assessment,” including why only very high countries are selected as reference countries, what was the rationale for increasing the lower and upper thresholds with that magnitude, and what was the process of selecting multiple criteria for changing the lower threshold. The “balanced assessment” in Slovakia is directed by recommendations regarding national decision-making in France, Germany, Scotland, and England from the Haute Autorité de Santé, Gemeinsame Bundesausschuss, Scottish Medicines Consortium, and National Institute for Health and Care Excellence, respectively, or reimbursement status in these countries. It is worth mentioning that although the Federal Joint Committee (Gemeinsame Bundesausschuss) is the highest decision-making body of the joint self-governance of health insurance funds in Germany, it is not the agency responsible for the HTA of pharmaceuticals.

Inappropriateness of transferring HTA recommendations from other jurisdictions has been described in the scientific literature (Kaló et al., 2012; Drummond et al., 2015). Furthermore, the application of international HTA recommendations—which may not include important details, such as price discounts—in Slovakian reimbursement decisions has no scientific validity and thus does not improve the evidence base of pharmaceutical policy decisions.

The problem is further amplified by the fact that strategic pricing of innovative pharmaceuticals is not based on small markets with relatively low purchasing power, such as Slovakia. The application of a balanced assessment could be one of the reasons for postponing the submission of reimbursement dossiers in Slovakia. The submission of dossiers after positive recommendations or reimbursement listing in England, France, Germany, and Scotland may result in easier market access with potentially higher prices in Slovakia. It should be emphasized that the health status of Slovak citizens is significantly worse than that in Western European countries.

Huic et al. (2019) concluded that marketing authorization holders submitted reimbursement dossiers in Slovakia for only 10 out of 25 of the innovative pharmaceuticals approved by the European Medicines Agency in a 1.5-year period (01/01/2017–30/06/2018). It is important to emphasize that marketing authorization holders submitted reimbursement dossiers in Slovakia without information on the relative effectiveness and cost-effectiveness of eight pharmaceuticals, and such information was available for only two pharmaceuticals.

In Slovakia the “balanced assessment” system created an opportunity for pharmaceutical manufacturers to obtain reimbursement for new medicines without relative effectiveness and economic evaluations adapted to the Slovakian market, if the medicine was aimed to treat a disease for which the number of patients eligible, based on the indication approved in the marketing authorization in the Slovak Republic, was lower than 1:50,000. This means that medicines that potentially do not show good value for money could be reimbursed through a fast-track pathway. Such exclusion of cost-effectiveness assessment has eased market access criteria not only for orphan drugs but also for heavily overpriced medicines, which treat subgroups of diseases, e.g., in the field of oncology.

Other elements of the 2018 legislation are also questionable. For example, the application of a higher threshold for medicines with a higher level of incremental QALYs *may result in the double counting of QALYs and discriminate medical prevention (e.g., vaccination programs) with a typically low health gain per patient*. Also, *extension of the time horizon in economic model may result in higher estimated QALY gain, which is rewarded by increased threshold, regardless of the associated uncertainty in health gain estimates with longer extrapolation period*.

A collaborative project between the Ministry of Finance and Ministry of Health of the Slovak Republic referred to as the Revision of Health Expenditure recommended an amendment to the reimbursement regulations to avoid overspending the pharmaceutical budget of the public health insurance system (Dancikova et al., 2018). The collaborative project concluded that—even if not all 59 reimbursement applications with fast track approval were added to the reimbursement list—the newly reimbursed medicines with partial assessment are increasingly consuming the public budget available for medicines with full assessment according to standard rules. It can be concluded that the legislation that came into force on January 1, 2018 was not financially sustainable for the Slovak Republic.

The legislation was changed from January 1, 2019 to allow accelerated reimbursement without pharmacoeconomic analysis for orphan drugs alone, to the exclusion of medicines that treat subgroups of diseases with a prevalence below 1:50,000. In addition, the updated legislation mandated only a 25% price reduction (instead of the 30% initial price reduction in 2018) to the first biosimilar in the Slovak market, compared to the price of the reference biological drug.

## Conclusions

HTA has become a standard policy tool used to inform decision-makers who must manage the entry and use of pharmaceuticals, medical devices, and other technologies (including complex interventions) within health systems, for example, through reimbursement and pricing. Many good practices have been developed in the areas of assessment and other key aspects of defining HTA processes (Kristensen et al., 2019).

Slovakia has limited financial resources to reimburse pharmaceutical technologies. Therefore, the concept of applying HTA prior to the pricing and reimbursement of new medicines may be even more important for Slovakia than for more affluent Western European countries. The HTA is still in a relatively early stage of implementation in Slovakia. However, Slovakian health insurance funds advocate the adoption of HTA. The efficiency of HTA processes in Slovakia can be improved by applying tools developed by the European network for HTA (EUnetHTA), such as the standardized submission template, the HTA Core Model^®^, and Methodological Standards for HTA2. Tesar et al. (2017) concluded that further legislative activities and reuse of relative effectiveness assessments produced by EUnetHTA are required in Slovakia as a result of the approved strategy for EU Cooperation on Health Technology Assessments.

In 2018, other legislative changes related to HTA were made, as the market access criteria for new medicines were softened in Slovakia. Changes in the regulation—especially the application of international HTA recommendations without assessment of transferability—were not based on scientific rationale and were implemented without prior validation. With the reimbursement of many new medicines, pharmaceutical spending started to increase in 2018, and even further increases could be expected in subsequent years. Innovative pharmaceutical manufacturers have benefited from easier market access due to a higher cost-effectiveness threshold or even the elimination of effectiveness and cost-effectiveness criteria for medicines with less than 1:50,000 eligible patients.

Moreover, the sustainability of the pharmaceutical budget has not been supported by the increased utilization of more affordable generic and biosimilar medicines, as market access criteria for off-patent pharmaceuticals have become more complicated. Indeed, significant problems with the availability of biosimilars on the Slovak pharmaceutical market were documented previously, as only 14 biosimilar dossiers had been submitted for reimbursement between 2006 and September 2018 out of the 47 biosimilars with EMA approval (Golias, 2018). Scarce information about the availability of generics in Slovakia represents limitation of our study, which can be alleviated by results of an ongoing analysis.

Overall, public pharmaceutical expenditure has become unsustainable, and the implementation of a “balanced assessment” has failed in Slovakia. Consequently, the legislation had to be changed on January 1, 2019.

## Data Availability Statement

All datasets generated for this study are included in the manuscript and/or the supplementary files.

## Author Contributions

TT, BO, ZK, and FK conceived the conception and design of the study. TT and BO contributed in acquisition of data. TT prepared the draft of the manuscript. All authors contributed to editing the manuscript, and the approved final version was submitted for publication.

## Funding

The article processing fee will be covered by the department of Organisation and Management in Pharmacy, Faculty of Pharmacy, Comenius University in Bratislava, Slovakia.

## Conflict of Interest Statement

The authors declare that the research was conducted in the absence of any commercial or financial relationships that could be construed as a potential conflict of interest.

## References

[B1] Barnieh L. Manns B. Harris A. Blom M. Donaldson C. Klarenbach S. (2014). A synthesis of drug reimbursement decision-making processes in organisation for economic co-operation and development countries. Value Health 17, 98–108. 10.1016/j.jval.2013.10.008 24438723

[B2] Bucek Psenkova M. Visnansky M. Mackovicova S. Tomek D. (2017). Drug policy in Slovakia. Value Health Reg. Issues 13, 44–49. 10.1016/j.vhri.2017.07.002 29073987

[B3] Dancikova Z. Grajcarova L. Kozak D. Marek A. Slobodnikova S. Stofko M. (2018). Revision of health expenditure II. [Interim report]. Ministry of Finance of the Slovak Republic Available online at: https://www.finance.gov.sk/sk/financie/hodnota-za-peniaze/revizia-vydavkov/revizia-vydavkov.html (Accessed March 29, 2019).

[B4] Dankó D. (2014). Health technology assessment in middle-income countries: recommendations for a balanced assessment system. J. Mark. Access Health Policy 2, 10.3402/jmahp.v2.23181 PMC4865748 27226832

[B5] Drummond M. Augustovski F. Kaló Z. Yang B. M. Pichon-Riviere A. Bae E. Z. (2015). Challenges faced in transferring economic evaluations to middle income countries. Int. J. Technol. Assess. Health Care 31, 6 442–448. 10.1017/S0266462315000604 26831815

[B6] Golias P. (2018). Analysis and options for wider use of biosimilar treatment in Slovakia. Institute for Economic and Social Reforms, Bratislava, Slovakia Available from: http://www.ineko.sk/clanky/biosimilarne-lieky-mozu-usetrit-desiatky-milionov-eur (Accessed May 14, 2019).

[B7] Huic M. Lipska I. Tesar T. (2019). Making high value medicines available in the CEE: a survey of multiple CEE countries. Presentation at the symposium Accessibility & impact of high value medicines, London Available from: https://www.accessibility-symposium.org/ (Accessed March 29, 2019).

[B8] Kaló Z. Docteur E. Moïse P. (2008). Pharmaceutical pricing and reimbursement policies in Slovakia. OECD Health Working Papers 31, 1–54. 10.1787/244264621247

[B9] Kaló Z. Landa K. Vokó Z. (2012). Transferability of NICE recommendations for pharmaceutical therapies in oncology to Central-Eastern European countries. Eur. J. Cancer Care (Engl.) 214, 442–449. 10.1111/j.1365-2354.2012.01351.x 22510226

[B10] Kaló Z. Annemans L. Garrison L. P. (2013). Differential pricing of new pharmaceuticals in lower income European countries. Expert Rev. Pharmacoecon. Outcomes Res. 13, 735–741. 10.1586/14737167.2013.847367 24219049

[B11] Kristensen F. B. Husereau D. Huic M. Drummond M. Berger M. L. Bond K. (2019). Identifying the need for good practices in health technology assessment: summary of the ISPOR HTA Council Working Group Report on Good Practices in HTA. Value Health 22, 13–20. 10.1016/j.jval.2018.08.010 30661627

[B12] Ministry of Health (2011). [Act No. 363/2011 Coll. on the scope and conditions of payments for medicines, medical devices and dietetic foods from public health insurance and amending certain acts, as amended] Zákon č. 363/2011 Z. z. o rozsahu a podmienkach úhrady liekov, zdravotníckych pomôcok a dietetických potravín na základe verejného zdravotného poistenia a o zmene a doplnení niektorých zákonov. Available online at: http://www.zakonypreludi.sk/zz/2011-363 (Accessed March 29, 2019).

[B13] Ministry of Health (2018). [Decree No. 93/2018 of the Ministry of Health of the Slovak Republic on the criteria for determining the significance of the effect of a medication on the public health insurance funds budget, the evaluation criteria for the calculation of the threshold value coefficient, and the details of the calculation of the threshold value coefficient] Vyhláška č. 93/2018 MZ SR o kritériách na stanovenie významnosti vplyvu lieku na prostriedky verejného zdravotného poistenia, o hodnotiacich kritériách pre výpočet koeficientu prahovej hodnoty a o podrobnostiach výpočtu koeficientu prahovej hodnoty. Available online at: http://www.epi.sk/zz/2018-93 (Accessed March 29, 2019).

[B14] OECD/EU (2018). Health at a glance: Europe 2018: state of health in the EU cycle, OECD Publishing, Paris 10.1787/health_glance_eur-2018-en. 10.1787/health_glance_eur-2018-en

[B15] Psenkova M. Mackovicova S. Tomek D. (2014). Impact of introducing costs/Qaly threshold on access to oncology medicines in Slovakia. Value Health 17 (7), A654–5. 10.1016/j.jval.2014.08.2387 27202368

[B16] Smatana M. Pažitný P. Kandilaki D. Laktišová M. Sedláková D. Palušková M. (2016). Slovakia: health system review. Health Syst. Transit. 18, 1–210. http://www.euro.who.int/__data/assets/pdf_file/0011/325784/HiT-Slovakia.pdf?ua=1 (Accessed May 10, 2019). 28139461

[B17] Szalay T. Pažitný P. Szalayová A. Frisová S. Morvay K. Petrovič M. (2011). Slovakia: health system review. Health Syst. Transit. 13 (2), 1–200. http://www.euro.who.int/__data/assets/pdf_file/0004/140593/e94972.pdf (Accessed May 10, 2019). 21540135

[B18] Tesar T. Hloska A. Wawruch M. Lehocka L. Snopkova M. Masarykova L. (2017). Introduction of health technology assessment for medicines in Slovakia. Int. J. Technol. Assess. Health Care 33, 345–349. 10.1017/S026646231700006X 28434416

[B19] Van Wilder P. Mabilia V. Kuipers Cavaco Y. McGuinn J. (2015). Towards a harmonised EU assessment of the added therapeutic value of medicines. Study of the ENVI Committee Available online at: http://www.europarl.europa.eu/RegData/etudes/STUD/2015/542219/IPOL_STU(2015)542219_EN.pdf (Accessed March 29, 2019).

[B20] Vogler S. (2012). The impact of pharmaceutical pricing and reimbursement policies on generics uptake: implementation of policy options on generics in 29 European countries—an overview. GABI J. 1 (2), 93–100. 10.5639/gabij.2012.0102.020

